# Rethinking STI control strategies: epidemiological and social determinants insights from a combined ecological and cross-sectional study in a Brazilian capital

**DOI:** 10.1186/s12889-025-23589-0

**Published:** 2025-07-03

**Authors:** Paula Knoch Mendonça Gil, Alisson André Ribeiro, Camila Guadeluppe Maciel, Márcio José de Medeiros, Cláudia Du Bocage Santos-Pinto, Everton Falcão de Oliveira

**Affiliations:** 1 Programa de Pós-Graduação em Doenças Infecciosas e Parasitárias, Universidade Federal de Mato Grosso do Sul, Campo Grande, Mato Grosso do Sul Brasil; 2 Faculdade de Engenharias, Arquitetura e Urbanismo e Geografia, Universidade Federal de Mato Grosso do Sul, Campo Grande, Mato Grosso do Sul Brasil; 3 Secretaria Municipal de Saúde, Campo Grande, Mato Grosso do Sul Brasil; 4 Centro Multidisciplinar UFRJ-Macaé, Instituto Politécnico, Universidade Federal do Rio de Janeiro, Macaé, Rio de Janeiro, Brazil; 5 Faculdade de Medicina, Universidade Federal de Mato Grosso do Sul, Campo Grande, Mato Grosso do Sul Brasil

**Keywords:** Sexually transmitted infections, Pre-Exposure prophylaxis, Disease reporting, Health profile, Hepatitis B virus, Syphilis

## Abstract

**Background:**

Sexually transmitted infections (STIs) are associated with substantial adverse outcomes, including genital symptoms, pregnancy complications, infertility, increased risk of HIV transmission, and significant psychosocial impacts. Identifying priority areas for action and key elements to inform discussions on expanding access to STI prevention measures, including HIV pre-exposure prophylaxis (PrEP), is essential for planning effective control strategies.

**Methods:**

This ecological study aimed to analyze the occurrence of notifiable STIs using data from a medium-sized state capital during the five years preceding the introduction of PrEP. Data on confirmed cases of notifiable STIs (HIV/AIDS, syphilis, and viral hepatitis) reported to the Brazilian Notifiable Diseases Information System (SINAN) in Campo Grande from 2014 to 2018, along with socioeconomic and demographic data, were assessed. Spatial analysis methods were used to identify clusters and areas with increased risk of STI occurrence.

**Results:**

A total of 10,074 STI cases were reported to SINAN. Syphilis was the most frequently reported infection, accounting for 78.6% of cases, followed by HIV/AIDS (19.5%) and hepatitis B (1.9%). Higher risk for all three STIs was observed among non-white men over the age of 20 low educational attainment. Among HIV cases specifically, heterosexual individuals were the most affected, based on available data regarding sexual orientation. Spatial analysis revealed clusters of high incidence in peripheral neighborhoods of the city. Additionally, correlation analysis indicated an association between STI occurrence and lower socioeconomic conditions.

**Conclusions:**

These findings suggest that STI control strategies should be re-evaluated to enhance coverage among individuals with the sociodemographic profile identified in this study, underscoring the need to broaden prevention strategies beyond traditionally defined key populations.

**Supplementary Information:**

The online version contains supplementary material available at 10.1186/s12889-025-23589-0.

## Background

Sexually transmitted infections (STIs) continue to pose a significant global public health challenge, with more than one million new cases acquired daily in 2020 [1]. According to the latest estimates from the World Health Organization (WHO), approximately 374 million new infections of four easily treatable STIs– chlamydia, gonorrhea, syphilis, and trichomoniasis– were reported worldwide in 2020. In the Americas, an estimated 38 million sexually active individuals aged 15–49 were affected during the same period [1].

Analyses from the Global Burden of Disease Study 2019 [2] revealed a decrease in age-standardized rates of incidence and disability-adjusted life years (DALYs) associated with STIs in several countries [3]. However, despite these reductions, the absolute number of incident cases and DALYs has increased over the past three decades (1990–2019) [3]. The greatest burden continues to fall on low- and middle-income countries, particularly in sub-Saharan Africa and Latin America, whereas higher-income regions have achieved significant progress, especially in HIV prevention, due to better access to effective interventions [3].

STIs are associated with substantial health consequences, including genital symptoms, pregnancy complications, infertility, increased risk of HIV transmission, and adverse psychosocial impacts [1]. To address these challenges, the WHO has renewed its Global Health Sector Strategy for the prevention and control of STIs for the 2022–2030 period [4]. The strategy emphasizes a combination of shared and disease-specific actions at the country level, supported by initiatives led by the WHO and its partners. Notably, reducing STIs remains a critical component of HIV prevention efforts and sexual and reproductive health programs. Additionally, the strategy highlights the importance of strengthening the capacity to monitor STI trends over time [4, 5].

In Brazil, the promotion of combined prevention is a cornerstone of the Ministry of Health’s strategy to control STIs. This comprehensive approach encompasses HIV pre-exposure prophylaxis (PrEP); regular STI testing to ensure timely diagnosis and appropriate treatment; HIV/AIDS post-exposure prophylaxis (PEP); consistent and correct condom use; harm reduction strategies; risk and vulnerability management; viral suppression through antiretroviral therapy; immunization programs; and the prevention of vertical transmission of HIV, syphilis, and hepatitis B [6, 7]. In accordance with national surveillance guidelines, the notifiable STIs in Brazil include HIV/AIDS, acquired syphilis, syphilis in pregnancy, and viral hepatitis B and C.

Brazil was the first country in Latin America to provide nationwide free access to PrEP, which began in early 2018 [8, 9]. Between 2018 and 2022, PrEP was exclusively recommended for four key populations: gay men and other men who have sex with men (MSM), transgender individuals, sex workers, and individuals in HIV-serodifferent partnerships [8]. In 2022, the PrEP protocol was updated to expand eligibility to all sexually active adults and adolescents (aged 15 and older) at increased risk of HIV/AIDS infection [7]. Despite these updates, we hypothesized that current prevention and control strategies, including PrEP provision, may still fall short of adequately covering all individuals at increased risk for STIs. Specifically, some populations– such as heterosexual individuals with low socioeconomic status– may not fit traditional key population definitions and therefore remain underserved by existing measures. This study was planned to explore whether such groups are being reached by current strategies and to inform the development of more inclusive public health actions.

To identify priority areas for action and support discussions on expanding access to STI prevention strategies, this study aimed to analyze the occurrence of newly diagnosed cases of notifiable STIs in a medium-sized state capital during the five years preceding the introduction of PrEP. Describing the epidemiological profile, analyzing the spatial distribution, and investigating the social determinants associated with the occurrence of these STIs is essential to understand the need for recognizing local specificities that should be considered when proposing public policies for STI control.

## Methods

### Study design, setting and period

This ecological study is based on data from confirmed cases of notifiable STIs (HIV/AIDS, syphilis, and viral hepatitis) in Campo Grande, Mato Grosso do Sul, Brazil, extracted from the Brazilian Notifiable Diseases Information System (Sistema de Informação de Agravos de Notificação, SINAN). Socioeconomic and demographic data were obtained from the 2010 Brazilian Census and from annual projections and estimates published thereafter. In 2014, the starting year of the data series analyzed in this study, Campo Grande had an estimated population of 843,120 inhabitants [10]. It has a total area of 8,118.4 km², with an urban perimeter of 359.03 km², divided into 74 neighborhoods that served as the units of analysis in this study.

All confirmed cases involving individuals aged 10 years or older were included to exclude cases in children and, consequently, those resulting from vertical transmission. The study focused on cases reported in residents of Campo Grande, Mato Grosso do Sul, between 2014 and 2018.

### Data cleaning, case definition, and frequency measures

The databases for the three STIs were reviewed to retain only cases reported for residents of Campo Grande, and were checked for duplicates and/or triplicates.

To clean the acquired and gestational syphilis databases, two criteria were applied: (1) the interval between notifications, with those having intervals of less than six months categorized as possible duplicates, and those with intervals of more than six months considered a potential reinfection or reactivation, thus classified as a new notification; (2) the interpretation of non-treponemal test results: titers of 1:4 or lower were classified as serological scars and excluded from new cases, while titers greater than 1:4 were classified as active infections and counted as new cases. In cases where non-treponemal test results were unavailable, the interval between notifications was used, i.e., criterion 1 [7, 11, 12]. Cases of acquired and gestational syphilis were combined into a single category (“syphilis cases”), considering that the recommendation of PrEP for pregnant women could provide benefits comparable to those observed in the general population [6, 8].

For viral hepatitis, only cases of hepatitis B (identified through the HBsAg marker) and co-infections were included, as the predominant mode of transmission for these cases is sexual. After data cleaning, the dataset was anonymized to ensure confidentiality before proceeding with the analyses, including geocoding procedures.

The analysis included sociodemographic variables (age group, sex, race/ethnicity, and education), data related to HIV/AIDS (specification of sexual orientation in HIV-cases linked to sexual transmission), and data related to hepatitis B (history of sexual contact with carriers of hepatitis B virus [HBV] or hepatitis C virus [HCV], drug use, and number of sexual partners).

To calculate the incidence/detection rate, annual population estimates provided by the Brazilian Institute of Geography and Statistics (Instituto Brasileiro de Geografia e Estatística, IBGE) [10] were used. The base population was composed of individuals aged 10 years or older. Mortality data were obtained from the Brazilian Mortality Information System (Sistema de Informação sobre Mortalidade, SIM).

### Socioeconomic variables and data sources

Socioeconomic and sanitation variables routinely used to assess social determinants of health were included, based on their availability for the study area. The following composite indices were analyzed:

- Education index: proportion of literate individuals aged 10 years or older.
- Income and poverty index: reflects economic conditions and income inequality, combining the average monthly household income (in minimum wages) with the proportion of households earning more than one minimum wage.
- Environmental sanitation index: measures urban infrastructure and access to services, including the percentage of households with piped water, sewage systems, in-house bathrooms, and garbage collection.
- Housing and living conditions index: captures housing adequacy, based on the proportion of owner-occupied homes, residents in permanent structures, and the average number of exclusive-use bathrooms in those households.
- Urban quality of life index: calculated as the average of the four indices above, summarizing overall urban living conditions.

These data, along with those used to calculate the frequency measures in this study, were obtained from publicly available governmental sources, including demographic, health, and socioeconomic datasets. A detailed list of data sources and access links is provided in the supplementary material [see Additional file 1].

### Statistical analysis

The study analysis was divided into two steps: in the first one, a descriptive analysis was carried out and all cases residing in Campo Grande were considered. In the second one, spatial analysis was applied and only cases with a geocoded street address were included.

Frequency distributions, as well as calculations of mean, median, standard deviation (SD), and proportions, were performed. The measure of infection frequency used was the incidence/detection rate per 100,000 inhabitants. For cumulative incidences, the total number of new cases over the five-year period was used, divided by the population of the median year in the time series, which was 2016. For deaths, the measures used were the case fatality rate and the cause-specific crude mortality rate.

The chi-square test or Fisher’s exact test were applied to verify whether there was a statistical difference between the frequencies by category of the socioeconomic ad demographic data and the three STI.

After the general characterization, STI cases were spatially mapped and grouped by neighborhoods. Spearman’s rank correlation coefficient was used to evaluate the relationship between STI incidence and socioeconomic and demographic data at the neighborhood level. The results of this analysis were presented in a correlation and scatterplot matrix. A significance level of 5% (α = 0.05) was adopted.

The geocoding of the cases was performed using R software version 4.3.0 (http://www.r-project.org/) and the *tidygeocoder* package [13], which generated a shapefile of points. Some residential addresses from the original SINAN database could not be geocoded, resulting in these cases being assigned to the city’s centroid; these points were subsequently excluded from the analysis. The original database contained 10,077 points, and after correction, the final number of points was 9,950.

To estimate the global spatial autocorrelation of the cases, we applied Moran’s I [14], and to identify clusters, we used LISA (Local Indicators of Spatial Autocorrelation) [15]. Spatial autocorrelation measures the degree of similarity between values separated by a given distance, such that in a non-random phenomenon, values closer in proximity are expected to be more similar.

The global spatial correlation, measured by Moran’s I, is used to test the null hypothesis of spatial randomness, with the alternative hypothesis suggesting the presence of clusters. While Moran’s I indicates the presence or absence of clusters, it does not identify their locations. To address this, we used Local Indicators of Spatial Autocorrelation (LISA), specifically local Moran’s I and local Geary’s C, to locate clusters and assess their statistical significance. These analyses of both local and global spatial autocorrelation were performed using GeoDa [16].

To assess the spatial density of STI cases (points), we applied the Kernel Density Estimation (KDE) method. This approach estimates density using a circular window that moves through the data, calculating the number of points within a specified radius and applying a chosen function. A 2-kilometer radius and a Quartic function were used for this analysis. The KDE was performed using QGIS (https://www.qgis.org) and the Heatmap tool (kernel density estimation), which generated heatmaps illustrating the clustering of points.

This analysis differs from the Spatial Autocorrelation approach because, instead of using incidence values per district, we used the actual geolocations of the STI cases as input. While the LISA analysis normalizes clusters by population size, the KDE identifies clusters based on the absolute number of cases.

All maps are presented in the SIRGAS 2000 datum and UTM 21 S projection.

## Results

A total of 12,104 STI cases were reported to SINAN. After database review, 10,068 cases were included in the study. Syphilis was the most commonly reported disease, accounting for 78.6% of the total reports ($\:n$ = 7,909), followed by HIV/AIDS with 19.5% ($\:n$ = 1,967) and hepatitis B with 1.9% ($\:n$ = 192). Co-infections with other STIs were identified in 2.4% ($\:n$ = 191) of syphilis, 9.7% ($\:n$ = 191) of HIV/AIDS, and 5.2% ($\:n$ = 10) of hepatitis cases.

**Fig. 1:**
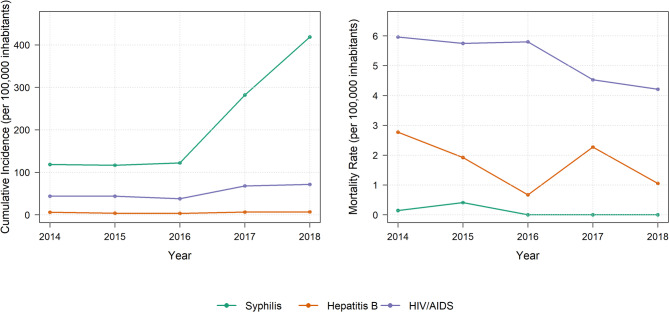
Annual cumulative incidence and cause-specific mortality rates of syphilis (acquired and gestational), viral hepatitis (HBV and co-infections), and HIV/AIDS per 100,000 inhabitants

The cumulative incidences for the period 2014–2018 were 1,058, 265, and 27 subjects per 100,000 inhabitants for syphilis, HIV/AIDS, and hepatitis B (either isolated or in co-infection with other forms of viral hepatitis), respectively. The annual temporal trends in incidence are presented in Fig. 1. Syphilis and HIV/AIDS reached their highest incidences in 2018, with a steady increase observed from 2016 onward. Hepatitis B showed its highest incidence in 2017 and 2018, with a continuous but comparatively smaller rise in cases from 2016, relative to the trends observed for syphilis and HIV/AIDS.

Hepatitis B exhibited the highest lethality among the STIs, with a rate of 10.5% (64 deaths), followed by HIV/AIDS at 9.8% (194 deaths) and syphilis at 0.1% (4 deaths). Over the study period, HIV/AIDS consistently recorded the highest STI-specific mortality rates, followed by viral hepatitis and syphilis, as illustrated in Fig. 1.

The mean age of syphilis cases was 31.5 years (median = 27, SD = 13.4), with the highest frequency observed in the 18 to 29-year age group (51.2%). Women accounted for the majority of cases (54.3%), as did non-white individuals (51.8%), and those with elementary education as their highest level of schooling (30.2%) (Table 1).

**Table 1:** Sociodemographic characteristics of syphilis, HIV/AIDS, and hepatitis B cases

Variable	Syphilis ($\:\varvec{n}\:$= 7,909)			HIV/AIDS ($\:\varvec{n}$ = 1,967)			Hepatitis B ($\:\varvec{n}$ = 192)			Total ($\:\varvec{n}$ = 10,068)		
	$\:\varvec{n}$	%	*p*-value	$\:\varvec{n}$	%	*p*-value	$\:\varvec{n}$	%	*p*-value	$\:\varvec{n}$	%	*p*-value
**Age (years)**												
10 to 17	508	6.4	< 0.001	35	1.8	< 0.001	10	5.2	< 0.001	553	5.5	< 0.001
18 to 29	4,050	51.2		826	42.0		25	13.0		4901	48.7	
30 to 39	1,571	19.9		536	27.2		26	13.5		2133	21.2	
40 to 59	1,401	17.7		486	24.7		97	50.5		1984	19.7	
60 and more	379	4.8		84	4.3		34	17.3		497	4.9	
**Sex**												
Women	4,296	54.3	< 0.001	476	24.2	< 0.001	76	39.6	< 0.05	4848	48.2	< 0.001
Men	3,612	45.7		1,491	75.8		116	60.4		5219	51.8	
Missing	1	0.0		0	0.0		0	0.0		1	0.0	
**Race/ethnicity**												
White	2,826	35.7	< 0.001	945	48.0	< 0.001	54	28.1	< 0.001	3825	38.0	< 0.001
Non-white	4,150	52.5		992	50.4		100	52.1		5242	52.1	
Missing	933	11.8		30	1.5		38	19.8		1001	9.9	
**Level education**												
Illiterate	33	0.4	< 0.001	3	0.2	< 0.001	3	1.6	< 0.001	39	0.4	< 0.001
Elementary school	2,390	30.2		668	33.9		64	33.3		3122	31.0	
High school	2,201	27.8		550	27.9		41	21.4		2792	27.7	
Higher education	692	8.7		403	20.5		10	5.2		1105	11.0	
Missing	2,593	32.8		343	17.4		74	38.5		3010	29.9	

For HIV/AIDS notifications, the mean age was 34.2 years (median = 32, SD = 12.1). Most cases occurred in individuals aged 18 to 29 years (42.0%), with men comprising 75.8% of cases. Individuals with elementary education represented the largest educational category (34.0%) (Table 1).

Hepatitis B cases had a mean age of 45.3 years (median = 47, SD = 15.3), with the 40 to 59-year age group accounting for 50.5% of cases. Men represented 60.4% of cases, while non-white individuals made up 52.1%. Similar to the other STIs, individuals with elementary education formed the largest group (33.3%) (Table 1).

Statistically significant differences were observed when comparing the internal frequency distribution (comparison between categories) of the variables presented in Table 1.

**Fig. 2:**
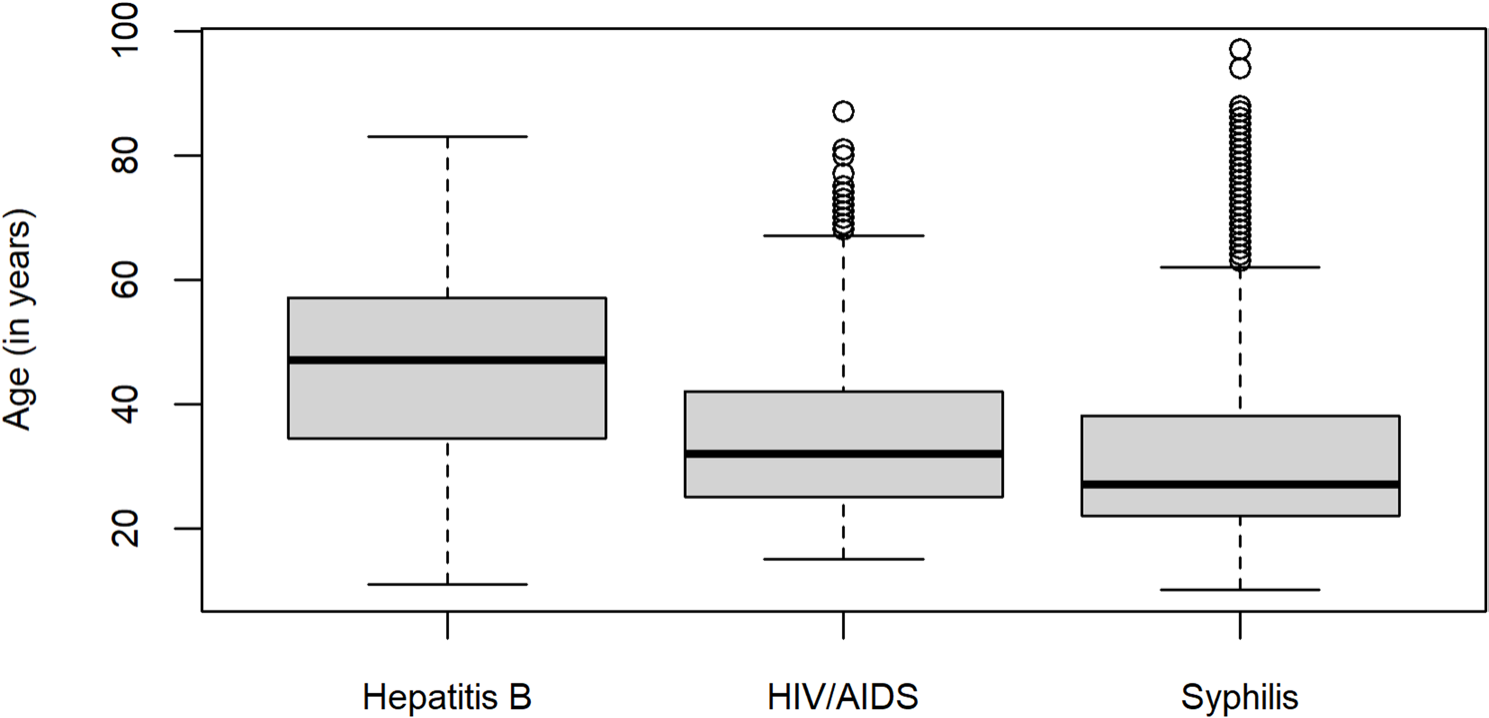
Age distribution of HIV/AIDS, hepatitis B, and syphilis cases

Based on these initial sociodemographic findings, a common profile of individuals at increased risk for STIs can be delineated: non-white men with up to 12 years of education (equivalent to completed high school). The only STI with a higher proportion of women was syphilis, which is likely explained by the systematic screening of pregnant women and subsequent reporting of gestational syphilis cases. This gender distribution aligns with established prenatal screening patterns. In terms of age, there is a notably higher risk of syphilis and HIV among adults aged 20 to 40 years, whereas hepatitis B is more frequent among individuals over 40. These patterns reflect distinct transmission routes and exposure histories across age groups. To illustrate these age-related differences, Fig. 2 presents a boxplot of the age distribution by infection type.

Among HIV/AIDS reports, the probable mode of transmission was predominantly sexual, accounting for 97.3% (1,915) of the reported cases. Of these, 55.3% (1,059) involved exclusively heterosexual relationships, while 35.7% (682) were associated with homo- or bisexual relationships. Parenteral transmission through the sharing of needles/syringes for injectable drug use was reported in 2.6% (51) of the total notifications.

For hepatitis B notifications, 95.8% (184) were isolated HBV infections, while 4.2% (8) involved co-infections with HCV or HAV. Co-infection with HIV was observed in 4.2% (8) of the cases. Prior sexual contact with individuals known to be positive for HBV or HCV was reported by 11.5% (22) of the cases, injectable drug use by 1.0% (2), and having three or more sexual partners in the past six months by 8.9% (17) of the cases.

**Fig. 3:**
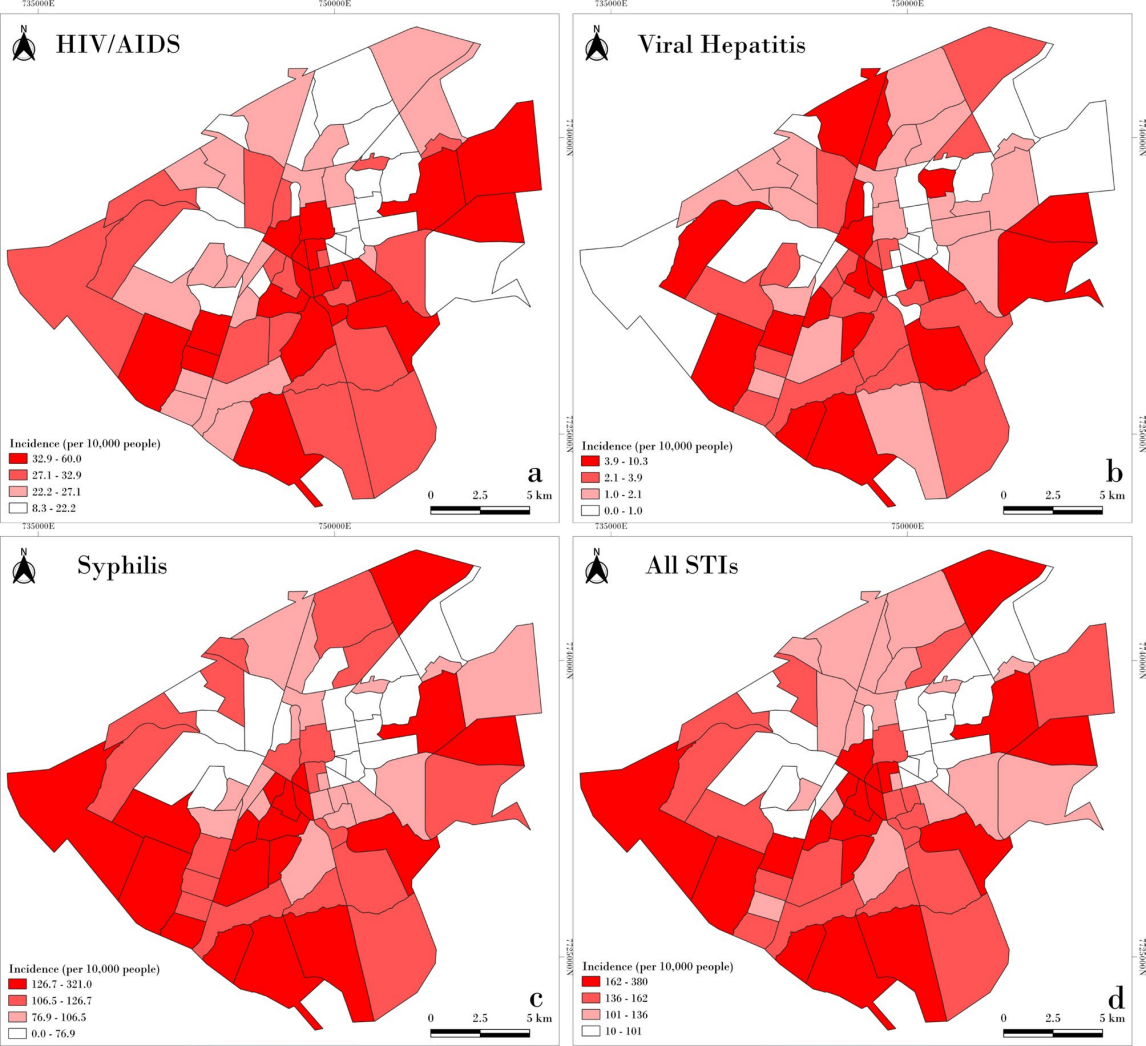
Spatial distribution of cumulative incidence of STIs according to neighborhoods in Campo Grande, Brazil, 2014–2018

**Fig. 4:**
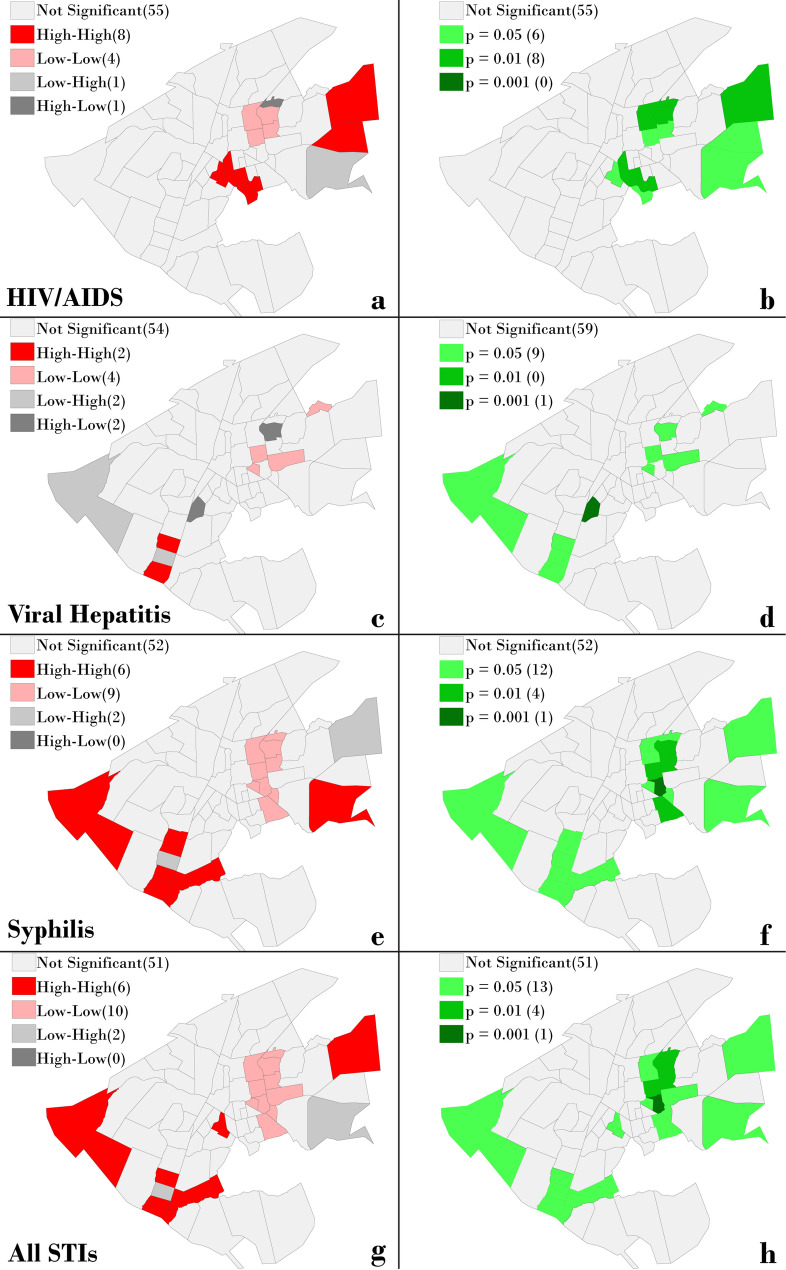
Local indicators of spatial autocorrelation (LISA) map for STIs

**Fig. 5:**
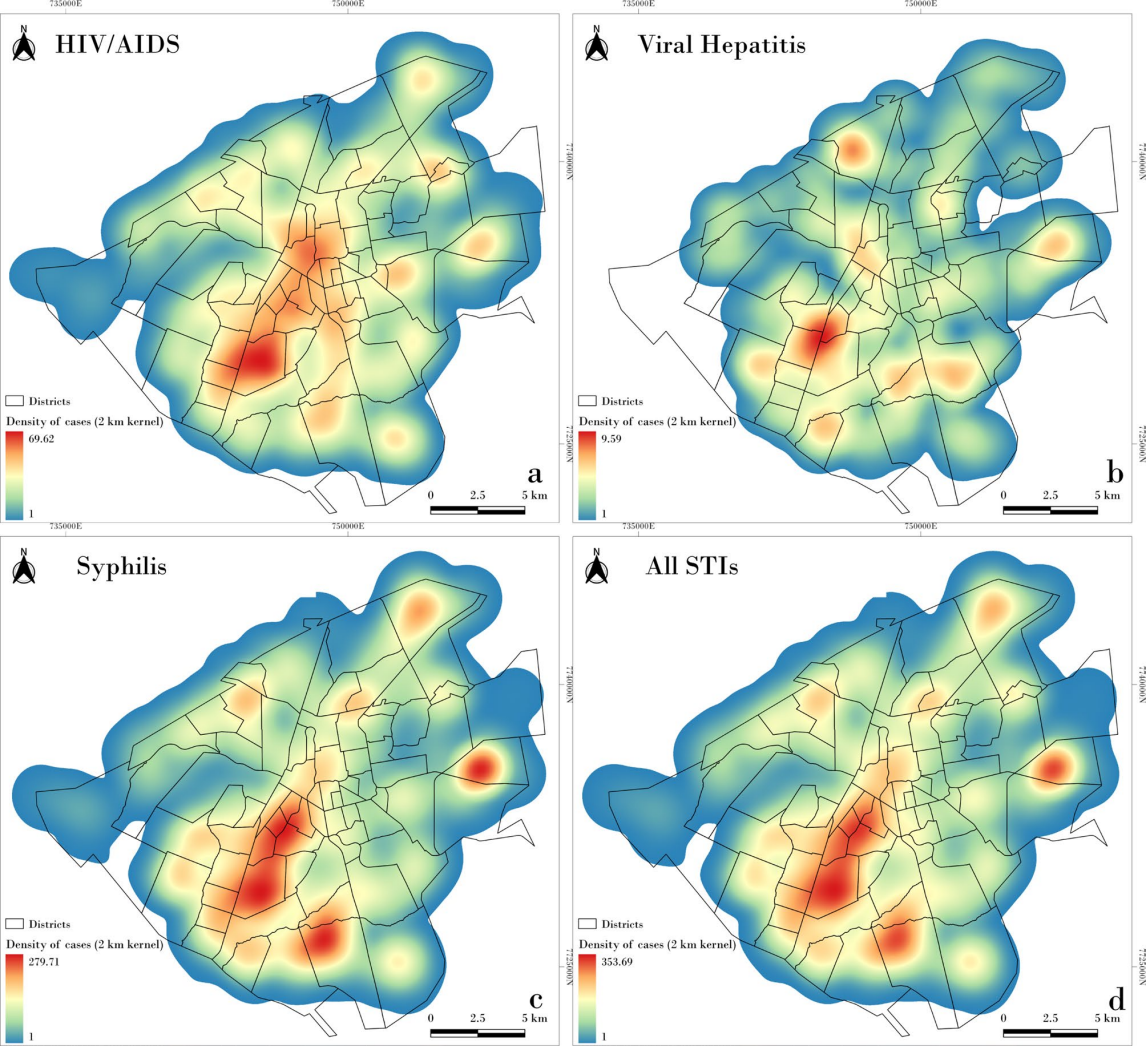
Heatmap of STIs cases generated using the Kernel Density Estimation (KDE) method

Figure 3 shows the spatial distribution of STI incidence by neighborhood. Descriptively, a similar pattern of occurrence is observed for all three STIs, with higher incidences concentrated in peripheral neighborhoods of the city. This pattern is supported by the LISA map analysis (Fig. 4), which reveals high-incidence clusters in the outskirts and low-incidence clusters in central neighborhoods. In the point-based cluster analysis (Fig. 5), similar aggregation patterns were observed, particularly for syphilis and hepatitis B, with case concentrations in the central-western region. Notably, a cluster of syphilis and overall STI cases was identified in the northeastern part of the city.

However, the spatial distribution of HIV cases showed some divergence between the two methods: while the LISA map (Fig. 4) suggests a concentration in the northeast, the point-based analysis (Fig. 5) indicates a higher density in the southwest. This discrepancy may be due to differences in the spatial resolution and nature of the datasets– LISA analysis was based on aggregated incidence by neighborhood, whereas the point analysis used individual case locations.

**Fig. 6:**
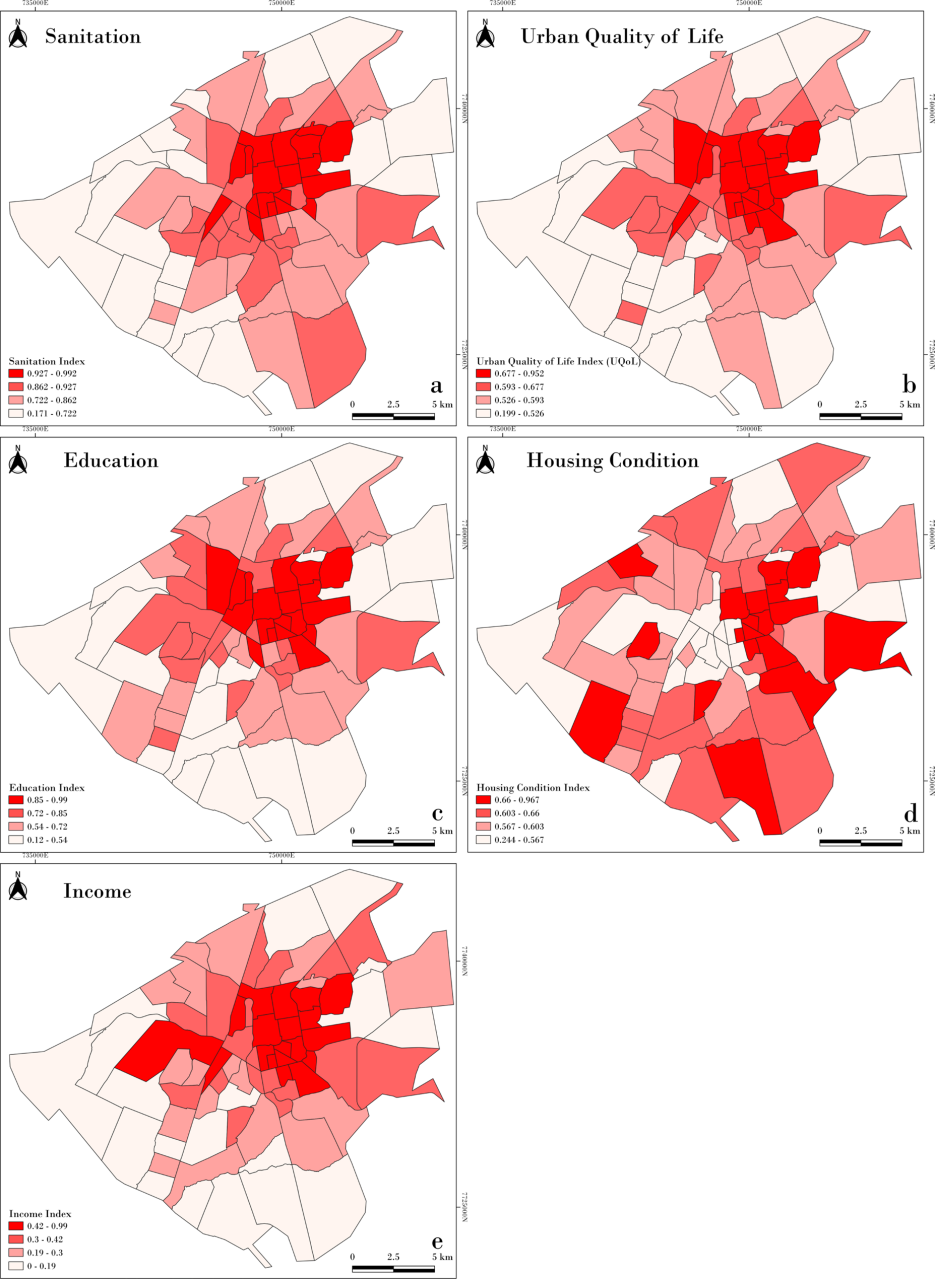
Spatial distribution of socioeconomic data by neighborhood

**Fig. 7:**
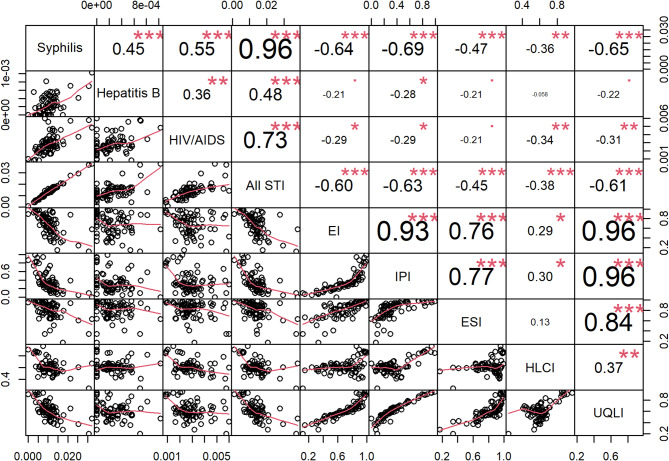
Scatter plots and matrix correlations of study data Legend: STI, sexually transmitted infectious; EI, education index; IPI, income and poverty index; ESI, environmental sanitation index, HLCI, housing and living conditions index; UQLI, urban quality of life index

Figure 6 illustrates the spatial distribution of socioeconomic data by neighborhood. Better sanitary conditions, urban quality of life indices, education, and income levels were predominantly observed in the central region of the study area. When assessing the correlation between these socioeconomic indicators and STI incidences, significant negative correlations were identified, particularly with income, urban quality of life, and education indices (Fig. 7). These findings suggest that the occurrence of these infections is associated with lower socioeconomic conditions. However, the housing condition index showed a distinct spatial pattern, diverging from the other indicators. One possible explanation is the high concentration of commercial establishments in the city center, which may result in fewer permanent residents, lower rates of homeownership, and reduced household-based data in those areas. As the index is based on the proportion of owner-occupied homes, residents in permanent structures, and the average number of exclusive-use bathrooms, these urban characteristics may distort the representation of residential living conditions in central neighborhoods.

## Discussion

Our results, combining individual and aggregate data analyses, revealed a profile of individuals at increased risk for STIs that should inform the planning and implementation of STI control strategies. Notably, these findings highlight the high incidence among individuals who do not fit traditional definitions of key populations [8], and who also require targeted approaches: men, heterosexuals, non-white individuals, with primary education, and aged up to 29 years, except for hepatitis B and co-infections, which showed higher incidence in the age group of 40 to 59 years. Although these are local data, our findings align with studies conducted in other Brazilian regions and internationally [3, 17–19].

Considering that regional and global trends in these infections are influenced by population changes, the methods employed in this study can be adapted to assess other settings on both small and large scales. These approaches can generate valuable data for controlling these infections and evaluating the impact and availability of interventions, such as vaccination; shifts in behaviors that alter STI transmission dynamics; and the role of international travel in spreading STIs in an increasingly interconnected world [20].

The interrelation between STIs and HIV, along with the substantially unequal burden of these infections globally and among key populations [20], must be taken into account. The rise in new HIV infections among adolescents and young adults should serve as a warning, highlighting the need for changes in the dissemination of information about STI prevention.

Despite many young individuals being exposed to prevention messages, they often fail to adopt preventive measures [21]. Tailoring educational approaches to the cultural context of this audience ensures more effective engagement. Brazilian initiatives like the School Health Program can leverage local partnerships and territorial specificities to address sexual and reproductive health with adolescents [22]. Sexual activity is beginning at increasingly younger ages and often occurs without protection. Implementing health education strategies targeting adolescents is crucial, particularly during puberty– a period marked by significant physical, emotional, and social changes, frequently accompanied by doubts and concerns typical of this stage of life [23–25].

In the context of sexual and reproductive education, combined prevention strategies for STIs must be integrated into the guidelines shared with adolescents, adults, and the elderly. In Brazil, since August 2022, PrEP– one of the most recent pillars of combined prevention methods– has been recommended for individuals aged 15 and older who are sexually active and at increased risk of HIV infection [6]. However, its coverage remains below the 50% target established by UNAIDS for men who have sex with men (MSM). Over 80% of current PrEP users in Brazil are MSM, indicating extremely low coverage among heterosexual individuals, a trend also noted in other studies [26, 27]. Furthermore, research has highlighted that the majority of individuals first learn about PrEP through the internet rather than from healthcare providers [28]. This underscores the importance of providing accurate, accessible, and easy-to-find online information about PrEP and HIV prevention. Internet-based advertising may serve as a promising channel for information dissemination and engagement [29].

Ultimately, our findings suggest that STIs disproportionately affect individuals experiencing adverse social determinants of health. Factors such as poverty, social marginalization, gender inequality, racial discrimination, unemployment, migration, and uneven health care coverage are consistently reported as contributors to the occurrence and persistence of STIs globally [30–32]. Additionally, the predominance of STIs among individuals who self-identified as non-white, as evidenced in other studies, may reflect barriers to accessing sexual and reproductive health services and combined prevention strategies for HIV and other STIs [19, 33, 34].

The spatial distribution of STI cases, particularly newly diagnosed HIV/AIDS cases, showed some discrepancies between area-based and point-based analyses. This may reflect differences in access to HIV testing and care services across the territory, which can influence where cases are detected. Further investigation into service availability and testing coverage could help clarify these patterns.

This context highlights the critical need to address structural barriers to accessing HIV prevention services in Brazil, particularly for both non-traditional and traditional key populations who continue to face challenges in accessing healthcare facilities. This is especially important considering that PrEP use and its associated follow-up require consistent engagement with the healthcare system [29]. These barriers include limited awareness of PrEP among both potential users and healthcare providers, stigma related to seeking HIV prevention services, and logistical challenges such as the limited availability of services in certain regions [26–29]. Investment in strategic health interventions tailored to the local epidemiological profile, such as targeting young heterosexual men and other populations eligible for PrEP, as well as efforts to improve vaccination coverage for hepatitis A and B, could enhance protection for healthcare users and society as a whole.

The primary limitation of this study lies in the reliance on data from information systems, where the completeness and quality of recorded data are beyond the researcher’s control, despite efforts to prevent information loss. The high frequency of missing data in certain fields, such as exposure category and sexual practices in notifications of HIV and viral hepatitis, may have impacted the findings. Another limitation of our study is that the spatial analysis was based on the place of residence, which may not reflect the actual location of STI acquisition. STIs often occur in contexts of mobility and social interaction beyond one’s neighborhood. Although surveillance data do not capture the likely place of exposure, using residence remains a useful proxy in ecological studies to identify socially vulnerable areas and guide prevention strategies. Nevertheless, the results provide valuable insights for analyzing and interpreting key aspects relevant to planning STI prevention strategies, particularly in the active search and tracking of sexual partners of incident cases.

This research provided valuable insights into the epidemiological characteristics of reported cases and the main provisions related to STI prevention, particularly concerning the implementation of PrEP in Campo Grande. If eligibility had remained restricted as before 2022, many individuals at higher risk of HIV infection would likely have been ineligible for this prophylaxis. The findings underscore the importance of planning targeted actions for key populations at greater risk of HIV and other STI, including expanding the dissemination of PrEP and other combined prevention strategies, training healthcare workers in updated protocols, and prioritizing services for these groups.

A comparison of the PrEP Clinical Protocols and Therapeutic Guidelines from the Brazilian Ministry of Health, published in 2018 and 2022, revealed an expansion in the populations eligible for this prophylaxis. Given that all individuals in this study presented exposure to STI-related risk factors, they could potentially benefit not only from PrEP as a biomedical strategy for HIV prevention but also from the broader package of care it entails. This includes regular testing, counseling, and access to combined prevention strategies, which are essential in the context of high syphilis prevalence and the co-occurrence of multiple STIs. Therefore, expanding access to integrated prevention services, such as those offered through PrEP programs, may represent a valuable opportunity to reach vulnerable populations and reduce the overall burden of STIs.

Considering this context, our findings, and the important role that primary healthcare units play within the Brazilian Unified Health System, we believe that strengthening primary healthcare services through expanded health education, broader access to STI testing, and extended clinic hours is a feasible short-term strategy for STI control. These initiatives could be implemented in existing family and community health units in Campo Grande, particularly targeting adolescents and heterosexual populations. Establishing measurable goals, such as increasing access to barrier methods and promoting early screening, would enhance the effectiveness of local STI prevention strategies.

## Conclusion

Understanding the epidemiological profile of populations affected by STI and its correlation with socioeconomic indicators as social determinants of health is essential for addressing and controlling these infections effectively. Our results highlight the need for specific interventions targeting groups not traditionally classified as key populations: adolescents and young adults, heterosexuals, and individuals with lower educational attainment. In addition to expanding targeted interventions for these groups, it is also crucial to adopt strategies that align with current realities, such as leveraging the internet and social media for awareness and outreach.

## Electronic supplementary material

Below is the link to the electronic supplementary material.

Supplementary Material 1

## Data Availability

All the data supporting the conclusions of this article are included in the article and its additional files.

## Abbreviations

IBGE: Brazilian Institute of Geography and Statistics
HBV: Hepatitis B virus
HCV: Hepatitis C virus
HIV/AIDS: Human immunodeficiency virus/ acquired immune deficiency syndrome
SINAN: Notifiable Diseases Information System
PrEP: Pre-exposure prophylaxis
STI: Sexually transmitted infections
WHO: World Health Organization
