# Supplementary material for: Rethinking STI control strategies: epidemiological and social determinants insights from a combined ecological and cross-sectional study in a Brazilian capital
Source: BMC Public Health. 2025 Jul 3;25:2314. doi: 10.1186/s12889-025-23589-0 (PMC12225224; doi:10.1186/s12889-025-23589-0)
Supplement: Supplementary file 1 — Supplementary Material 1 [file 12889_2025_23589_MOESM1_ESM.docx]

**Additional file 1**

**Data Sources**
The publicly available data used in this study can be accessed from the following repositories:

- Population estimates for Campo Grande (2014–2018):
  https://www.ibge.gov.br/estatisticas/sociais/populacao/9103-estimativas-de-populacao
- HIV/AIDS-related deaths: <https://indicadores.aids.gov.br/>
- Deaths from viral hepatitis: <http://indicadoreshepatites.aids.gov.br/>
- Deaths from acquired and gestational syphilis: <http://indicadoressifilis.aids.gov.br/>
- Socioeconomic data from the Campo Grande city profile:
  <https://cdn.campogrande.ms.gov.br/portal/prod/uploads/sites/18/2020/10/PERFIL-COMPLETO-PDF.pdf>
- Base map shapefile (public domain) for geospatial analysis:
  <https://www.ibge.gov.br/geociencias/downloads-geociencias.html>

Note: Health and IBGE data reports overlapped across the entire metropolitan region, which allowed for data comparability in the analyses.
